# Sex-specific vagal and spinal modulation of swallow and its coordination with breathing

**DOI:** 10.1371/journal.pone.0234194

**Published:** 2020-06-11

**Authors:** Alyssa Huff, Mitchell D. Reed, Kimberly E. Iceman, Dena R. Howland, Teresa Pitts

**Affiliations:** 1 Department of Physiology, University of Louisville, Louisville, Kentucky, United States of America; 2 Department of Neurological Surgery, Kentucky Spinal Cord Injury Research Center, University of Louisville, Louisville, Kentucky, United States of America; 3 Research Service, Robley Rex Veterans Affairs Medical Center, Louisville, Kentucky, United States of America; National Yang-Ming University, TAIWAN

## Abstract

Swallow-breathing coordination is influenced by changes in lung volume, which is modulated by feedback from both vagal and spinal sensory afferents. The purpose of this study was to manipulate feedback from these afferents, with and without a simultaneous mechanical challenge (chest compression), in order to assess the influence of each sensory pathway on swallow in rats. We hypothesized that manipulation of afferent feedback would shift the occurrence of swallow toward the inspiratory phase of breathing. Afferent feedback was perturbed by lidocaine nebulization, extra-thoracic vagotomy, or lidocaine administration to the pleural space in sodium pentobarbital anesthetized rats (*N* = 43). These different afferent perturbations were performed both in control conditions (no chest compression), and with chest compression. Manipulating pulmonary stretch receptor-mediated volume feedback in male animals decreased swallow occurrence. Female rats appear to rely more on spinal afferent feedback, as swallow occurrence shifted to late expiration with chest compression and vagotomy or lidocaine injections. Results suggest that sex-specific mechanisms modulate swallow-breathing coordination, and that vagal feedback is inhibitory to swallow-related muscles, while spinal feedback from pleural afferents has excitatory effects. This study supports the theory that a balance of vagal and spinal afferent feedback is necessary to maintain an optimal swallow pattern and swallow-breathing coordination.

## Introduction

The purpose of airway protection is to coordinate the passage of air into and out of the lungs and of foreign material into the esophagus. In 1789, Patten [1] described the first case of dysphagia (disorder of swallow), and in 1816 Magendie [2] described the three phases of swallow: oral, pharyngeal and esophageal. Kronecker and Meltzer in 1880 [3] discovered that swallow required the integration of brainstem respiratory centers with the activity of six cranial nerves [4], and they described swallow as the most complex “all or none” reflex. In 1887 Marckwald [5] and Wassilieff [6] identified a “swallow center” located in the 4^th^ ventricle of the brainstem of the rabbit and described the influence of swallow on breathing. In 1915, Miller and Sherrington [7] concluded that stimulation of many different medullary locations can elicit swallow. The possibility of spinal influences on swallow was supported by Sumi in 1963 [8], who reported that groups of medullary and spinal inspiratory and expiratory neurons were either excited or inhibited by swallow, even when the animals were paralyzed and artificially ventilated.

These pivotal studies form a foundation for the swallow field, and since then swallow has been studied *in vivo* in the mouse [9], rat [10, 11], bat [12], cat [13–19], rabbit [20, 21], pig [22], sheep [23], goat [24], monkey [25, 26], and human [27–31]. Swallow has also been studied *in situ* [19, 32–34] and *in vitro* [35] and modeled *in silico* [36, 37]. In humans, swallow has been studied mostly in populations with diseases including Parkinson’s disease [38–42], stroke [43, 44], and head and neck cancer [45].

Despite the progress that has been made in the last century to understand the complex behavior of swallow, our mechanistic understanding of this important behavior is relatively limited. Classically, swallow has been regarded as a brainstem-mediated behavior, but more recent studies have determined that afferent feedback is important in the coordination of swallow with breathing cycle [27, 29–31]. In the cat, swallow occurs in the late expiratory (E2) phase of the cough-breathing cycle [14], but upper abdominal laparotomy produces a significant shift of swallow to the inspiratory phase of the breathing cycle [46]. Several studies in the human demonstrate that—regardless if swallow occurs as single [29] or sequential events [30], with a thin or thick consistency bolus [29, 31], or if the system is challenged to coordinate with cough epochs [27]—swallow occurs during a targeted lung volume of 45–65% of vital capacity [27, 29–31]. In a previous publication, we reported this and developed the concept of lung *volume targeting*, which can explain swallow occurrence across any phase of cough in the human [27].

Lung volume regulation relies on both vagal and spinal afferent feedback, but the effects of these feedback sources on swallow occurrence is unknown. Seventy percent of heart-lung transplant patients are reported to develop oropharyngeal dysphagia [47], while up to 73% of patients suffering from a traumatic cervical spinal cord injury silently aspirate [48]. The mechanisms responsible for discoordination of swallow and breathing in these disease populations are not fully understood. In this study, we selectively reduced different types of afferent feedback with three different manipulations: vagotomy to eliminate all vagal sensory feedback, lidocaine nebulization to suppress vagal pulmonary stretch receptor (PSR) feedback, and lidocaine infusion into the pleural space to reduce spinal feedback from pleural afferents. Due to the strong evidence that PSR and other vagal feedback influences respiratory phase regulation, we hypothesized that loss of these important sensory feedback components would shift swallow occurrence more toward the inspiratory phase of the breathing cycle. Various studies have reported breathing-related sex differences such as respiratory rate [49], ventilatory responses to hypoxia and hypercapnia [49–51], prevalence of sleep apnea [52], cardiorespiratory homeostasis and neuroplasticity [53], and hormones (including progesterone, a respiratory stimulant) [54]. These differences lead us to suspect there may also be important sex-specific differences in swallow-breathing coordination.

## Methods

Experiments were performed on 48 anesthetized spontaneously breathing Sprague Dawley (SD) retired breeder rats [24 male (0.49 ± 0.04kg) and 19 female (0.39 ± 0.08kg), Envigo, Indianapolis, IN] of which only 43 completed the protocol. Ages ranged from 8–9 months. Animals were shared with a complementary paper to this study [55]. We recognize that retired breeders are older than the general adult rat, however studies investigating breathing and aging use SD rats with an average of 13 months [56]. The protocols were approved by University of Louisville Institutional Animal Care and Use Committee (IACUC). The animals were initially anesthetized with gaseous isoflurane (1.5–2% with 100% O_2_) while a femoral intravenous (i.v.) cannula was placed for administration of sodium pentobarbital (25 mg/kg, i.v.). Isoflurane was discontinued and supplementary doses of sodium pentobarbital were administered as needed throughout the experiment. Anesthetic level was evaluated by withdrawal reflex of the forelimb and hindlimb and licking in response to oral water administration. A dose of atropine sulfate (0.01mg/kg, i.v.) was given at the beginning of the experiment to reduce secretions from repeated tracheal stimulation. Following administration of atropine sulfate, a tracheostomy was performed and followed by incision into the esophagus to place a 20 gauge catheter to measure esophageal pressure. Body temperature was maintained using a heating pad. After completion of the experimental protocol, euthanasia was induced by an overdose of sodium pentobarbital followed by either administration of Beuthanasia D (Merck Animal Health) or potassium chloride.

Electromyograms (EMG) were recorded using bipolar insulated fine wire electrodes according to the technique of Basmajinan and Stecko [57]. Four muscles were used to evaluate swallow and/or breathing function: mylohyoid, geniohyoid, thyroarytenoid, and costal diaphragm. A small horizontal incision was made at the rostral end of the right digastric muscle exposing the surface of the mylohyoid and electrodes were placed in the right mylohyoid. A small horizontal incision was made on the rostral end of the left digastric continuing through to the left mylohyoid exposing the geniohyoid and electrodes were placed in the left geniohyoid. The thyroarytenoid electrodes were inserted through the cricothyroid window into the anterior portion of the vocal folds, which were visually inspected post-mortem. For electrode placement of the costal diaphragm, palpation and elevation of the xyphoid process was followed by insertion of a needle directly caudal, and the needle was hooked underneath the xyphoid process near the costal diaphragm muscle attachment. Electrodes were placed bilaterally into the pectoralis muscle to record electrocardiogram (ECG) activity, which was used to remove heart artifact from EMG traces.

### Defining respiratory phase

**Fig 1:**
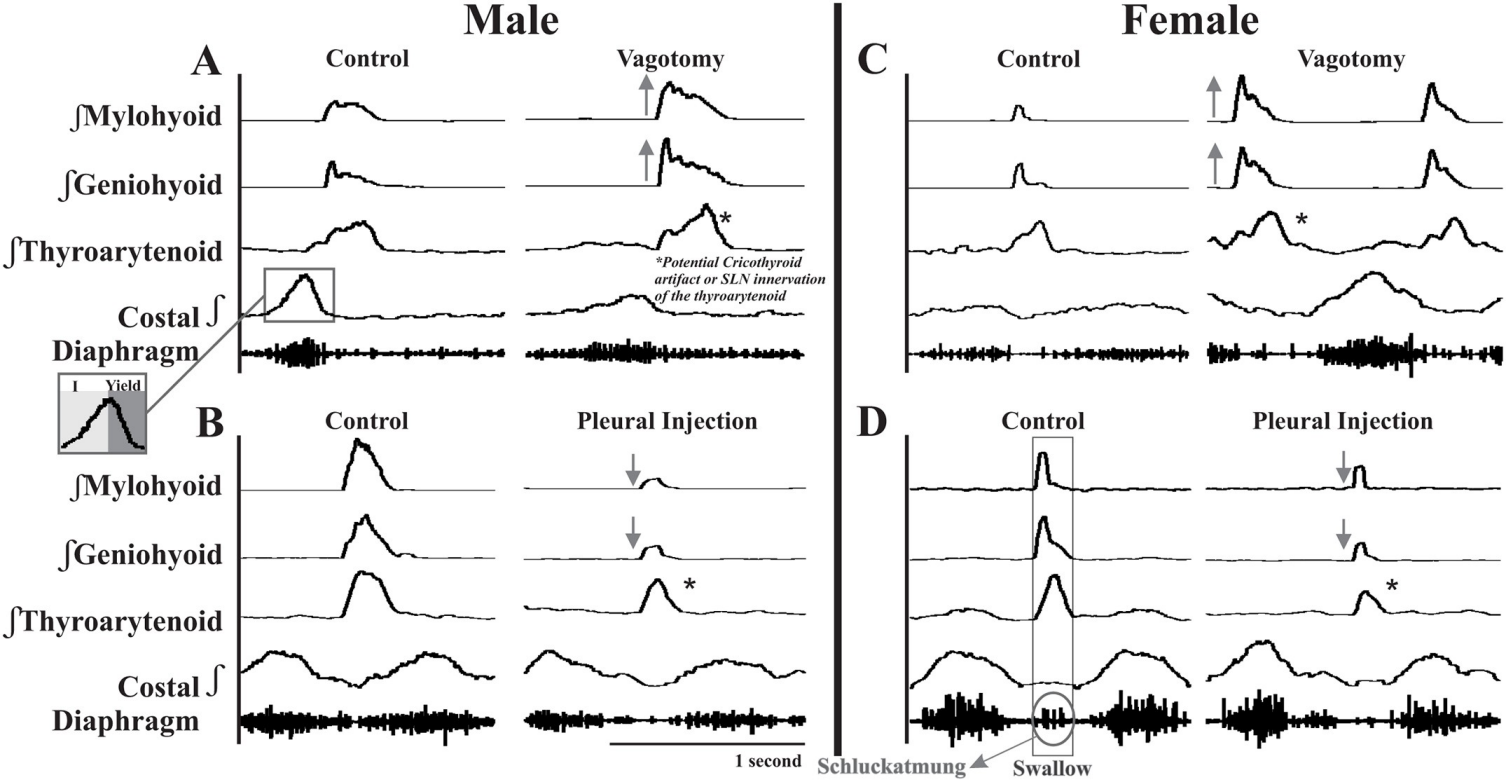
Representative EMG traces of swallow activity before and after afferent feedback manipulations. Panels A and B are recordings during swallows in male animals. Panels C and D are recordings in female animals. In both male and female animals, upper airway muscle amplitudes increase after vagotomy (A and C) and decrease after pleural injection (B and D). Panel A demonstrates the inspiratory and yield (remnant diaphragm activity in early expiration) components of breathing. Panel D displays schluckatmung (“swallow-breath”) diaphragm activation during swallow.

In the present study, inspiration (I) was defined as the period from the onset of diaphragm activity to the peak of the diaphragm burst, and expiration as the period from the peak of diaphragm activity to the onset of subsequent diaphragm activity (Fig 1). We defined vagal efferent activity that begins in early expiration as E1 (i.e. thyroarytenoid: laryngeal adductor), and the spinal inspiratory muscle activity remaining that remains during early expiration as “yield” (Fig 1). Late expiration (late E) was defined as the period from the offset of diaphragm activity to the onset of subsequent diaphragm activity. Yield is characterized by remnant diaphragm activity in early expiration that acts as a “cushion” to dampen forces from the chest wall onto the lungs. We derived this term from its use in locomotion studies, in which the term describes activation of knee and ankle extensor muscle to cushion the impact of forces on the body as the hips move over the knee [58]. The complementary paper to this study [55] presents a detailed description of this concept in respiration, and hypothesizes that characterizing early expiration as a yield event could aid in interpreting differences in late-I versus early-E activities of breathing.

### Experimental protocol

Three experimental protocols were performed on three cohorts of male and female SD rats. A) An extra-thoracic vagotomy was performed in 12 rats [6 male (0.48 ± 0.03kg) and 6 female (0.35 ± 0.06kg)]. B) Lidocaine (10%) was nebulized into the trachea in 18 rats [8 male (2 of which were sham protocol) (0.40 ± 0.03kg) and 10 female (2 sham)]. Only 5 of these females (2 of which are sham) completed the protocol (0.39 ± 0.09kg) and are included in the data presented. C) Lidocaine (10%) was injected into the pleural space in 18 rats [10 male (2 sham) (0.46 ± 0.05kg), 8 female (2 sham) (0.41 ± 0.08kg)]. In sham experiments for protocol B), saline (diluent) was nebulized into the trachea; for protocol C), saline was injected into the pleural space.

### Removal/Reduction of vagal feedback

A) To remove all vagal afferent feedback, bilateral vagotomy at the level of the extra-thoracic trachea was performed on rats in the supine position. The vagus nerves were dissected from the sympathetic nerves and common carotid arteries. Silk suture (5–0) was looped around each vagus nerve. Hemostat forceps were clamped onto the suture ends for quick access after control trials had been completed. At the appropriate time, the suture attached to the hemostats was lifted so that the vagus nerves could be cut using spring scissors at the level of the 5^th^– 6^th^ tracheal ring. After bilateral vagotomy, an inflation test was performed to assure removal of PSR (lung volume) feedback: 4 cc of air was drawn into a 5 cc syringe and quickly infused into the endotracheal tube. The order of the cuts were randomized (left vs right) across animals.
B) To selectively reduce vagal feedback from PSRs, 10% lidocaine was nebulized into the trachea with the animal in the supine position. Using a compressor nebulizer (Strong Health; particle size 0.5–5μm; average nebulization rate 0.2 mL/min), 10% Lidocaine (Cat No. L5647, Sigma-Aldrich) mixed with 2% Evans Blue Dye (EBD, Cat No. E2129, Sigma-Aldrich) was nebulized for 15 minutes. Ten minutes after the completion of the nebulization, we performed an inflation test by injecting 4cc of air into the trachea. If the Hering-Breuer reflex was maintained (i.e. termination of inspiration followed by prolonged expiration), suggesting that PSRs were not anesthetized, the animal then received an additional 5 minutes of nebulized lidocaine and was retested. This procedure was performed as necessary until the reflex was abolished. The addition of the dye allowed for post-mortem verification that the lidocaine penetrated the lung tissue and the intra- and extra-thoracic trachea. To minimize contamination of the lidocaine and dye into the air and to the researcher, a portable fume evacuation machine hovered over the mouthpiece of the nebulizer. To minimize contamination around the trachea, Vaseline-coated gauze was placed below and above the trachea, which covered any exposed area of the animal and blocked any potential absorption of lidocaine into untargeted areas.

### Reduction of spinal feedback

C) To reduce spinal feedback (via non-myelinated fibers of the peritoneum and the pleural space [59], as well as superficial mechano- and sensory receptors of the diaphragm), bilateral injections of 10% lidocaine mixed with 2% EBD were administered into the pleural space using methods from Mantilla et al. [60], which labeled motoneurons in both the cervical and thoracic segments indicating phrenic and intercostal innervation. Animals were stabilized and the rib cage was palpated to identify the fifth intercostal space. Each injection site was located one inch rostral to the xyphoid process and lateral to the sternum where the axial portion of the rib lies, and then marked with a permanent marker. At this location, the skin was removed using skin scissors, and 20μl of lidocaine/EBD mixture was injected bilaterally using a 100-μl Hamilton syringe with a 35 gauge beveled needle inserted 6 mm so that it reached into the pleural cavity. After both injections were complete, the animal was returned to supine position, and after a 5 minute waiting period an inflation test was performed to confirm that a reflex response was present, indicating that the lidocaine had not affected the PSRs or altered any other vagal afferent feedback.

#### Stimuli

Two stimuli were completed throughout various conditions in each protocol. Chest compression stimuli were performed during control conditions (before lidocaine or vagotomy interventions) and after interventions. Swallow stimuli were performed during control conditions (before lidocaine or vagotomy interventions) with and without chest compression, and during intervention conditions, both with and without chest compression.

Chest compression of the thoracic cavity was performed by placing a 2-inch thick circumferential Velcro band around the chest to restrict chest movement to the end of expiration tidal volume. In order to monitor movement of the chest wall, a custom in-house produced chest strap was made using a piezoelectric sensor from a fire alarm, an aluminum plate, and an elastic band (1/2 inch). The sensor was mounted on the aluminum plate, which was loosely strapped around the animal’s chest rostral to the Velcro restriction band. This piezoelectric chest strap allowed observation of the change in movement resulting from the restrictive band. Video was also taken for visual observation of the reduction in chest movement.

Swallow was induced by insertion of 1cc water into the oropharynx with a 1 inch long thin polyethylene catheter (diameter 2.37mm) attached to a 3 cc syringe. Swallow was defined as an activation of the mylohyoid, geniohyoid, and thyroarytenoid muscles and costal diaphragm, if present (representing the schluckatmung or swallow breath, Fig 1). Swallow stimuli were performed before and after intervention as well as during chest compression stimuli.

### Analysis

All EMG signals were amplified and filtered (100–1000 Hz) using Grass P511 (Natus Neurology) amplifiers. Esophageal pressure was measured by a TA-100 single channel transducer amplifier (CWE, Inc). Signals were rectified and integrated (20ms) using Spike2 (Cambridge Electronic Design; Cambridge, England). EMG amplitude measures were normalized to the largest swallow in the control trial with and without chest compression. The swallow parameters that were measured include total swallow duration (the period from the onset of mylohyoid activation to the offset of thyroarytenoid activation) and amplitudes of mylohyoid, geniohyoid, and thyroarytenoid muscles. The inactivity of the thyroarytenoid in conjunction with mylohyoid and geniohyoid activity distinguishes licking behaviors from swallow activity [25]. Thus, if thyroarytenoid activity was absent, the event was not included as a swallow.

Results are expressed as means ± standard deviation (SD). Paired t-tests and Wilcoxon signed ranks tests were used as appropriate to statistically identify differences using SPSS statistical software (IBM Corporation). Analyses were made within groups (male and female) and between groups (male vs female). A difference was considered significant if the *p*-value was less than or equal to 0.05.

## Results

### Swallow with chest compression

Injection of water into the oropharynx elicited an average of 6 ± 4 swallows in males and 9 ± 6 swallows in females during control conditions; chest compression did not change swallow number (Table 1).

**Table 1:** Means, Standard Deviation (SD), *p*-values, and direction of change for swallow parameters during control and chest compression conditions for both male and female groups. Amplitude is normalized to maximum of control and shown as a percentage.

	Control mean (SD)	Chest Compression mean (SD)	*p*-value	Change
**Male (*n* = 24)**				
Swallow Duration (ms)	296 (73)	303 (77)	0.64	**-**
Swallow Number	6 (4)	5 (4)	0.08	**-**
Mylohyoid Amplitude (% max)	72 (19)	111 (70)	**0.02**	**↑**
Geniohyoid Amplitude (% max)	78 (14)	110 (69)	**0.03**	**↑**
Thyroarytenoid Amplitude (% max)	87 (10)	115 (69)	*0*.*06*	↑
**Female (*n* = 19)**				
Swallow Duration (ms)	301 (93)	290 (70)	0.41	**-**
Swallow Number	9 (6)	7 (6)	0.17	**-**
Mylohyoid Amplitude (% max)	70 (19)	80 (41)	0.24	**-**
Geniohyoid Amplitude (% max)	76 (14)	85 (48)	0.39	**-**
Thyroarytenoid Amplitude (% max)	85 (10)	94 (21)	0.08	**-**

Reported *p*-values are from Student’s paired t-test. Significance is **bolded** at *p*-values ≤ 0.05 and *p*-values indicating trends toward significant of 0.05 < x ≤ 0.07 are *italicized*.

**Fig 2:**
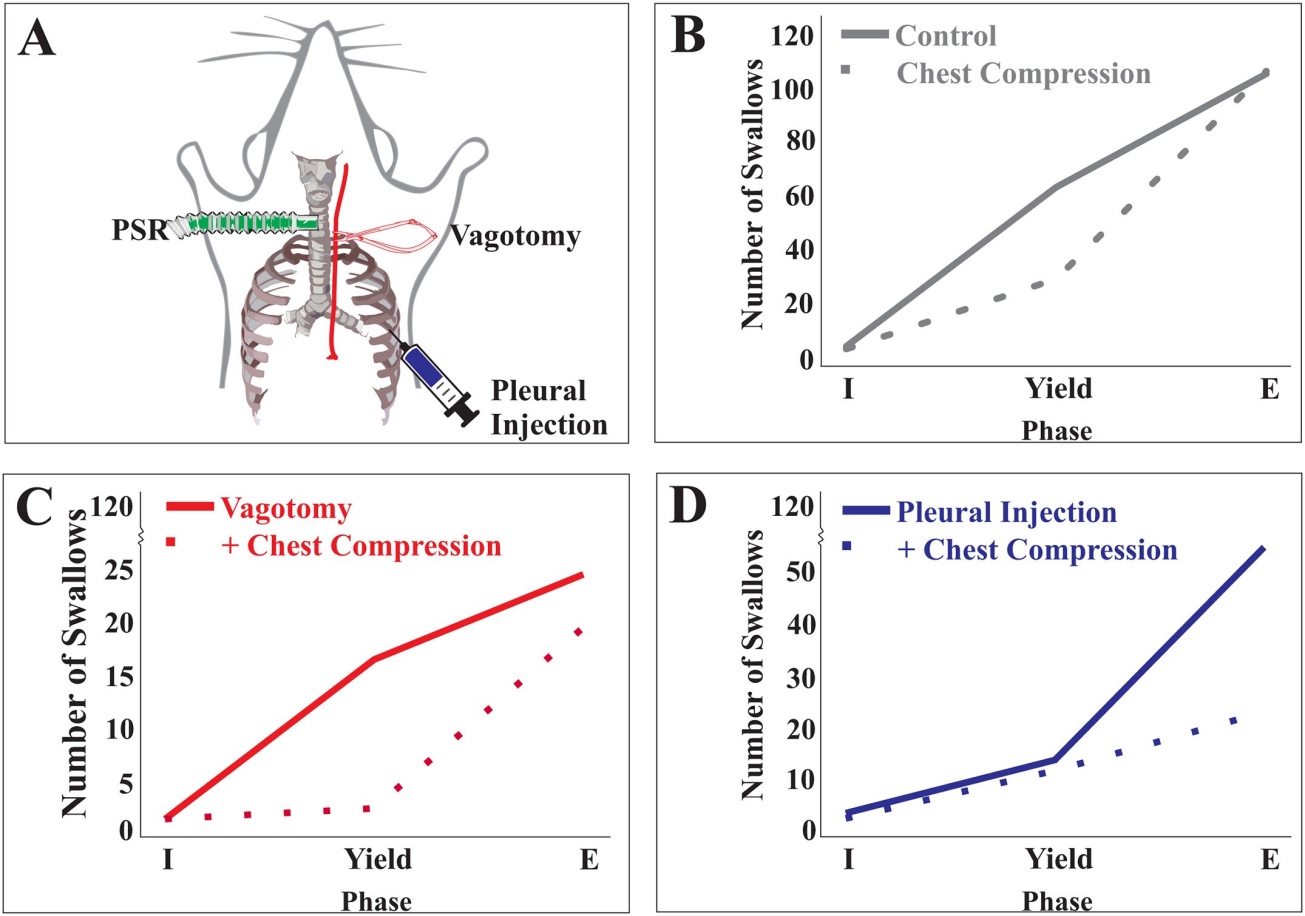
Experimental perturbations shifted swallow-breathing coordination in females. A) Illustrated representation of experimental protocol for afferent feedback manipulation. B) Chest compression shifted swallow-breathing coordination toward expiration, with swallow predominately occurring during expiration. In vagotomized females C), as well as those with reduced spinal feedback D), swallow-breathing coordination shifted toward expiration when chest compression was applied.

In control conditions, females produced 169 total swallows. Of those, 62% (104 of 169) occurred during late E, 37% (62 of 169) occurred during yield, and 1% (3 of 169) occurred during I. With application of chest compression, 135 swallows occurred, with 78% (105) occurring in late E, 21% (28) during yield, and 1% (2) during I. During chest compression, there was a significant shift in swallow-breathing phase preference, with more swallows occurring during late E (z = -3.2, *p* = 0.001; Table 2 and Fig 2b). Under control conditions, 154 swallows were elicited in males. Of those, 66% (101) occurred during late E, 32% (49) in yield, and 2% (4) in I; chest compression produced no significant change in swallow-breathing coordination (Table 2).

**Table 2:** Number of swallows during each phase of breathing for control and chest compression conditions for both male and female groups.

	Control # of Swallows	Chest Compression # of Swallows	*p*-value	Z
**Male**			0.99	-0.02
Inspiration (I)	4	8		
Yield	49	25		
Late Expiration (Late E)	101	85		
**Female**			**0.001**	-3.2
Inspiration (I)	3	2		
Yield	62	28		
Late Expiration (Late E)	104	105		

Reported *p*-values are from Wilcoxon signed ranks test. Significance is **bolded** at *p*-values ≤ 0.05 and *p*-values indicating trends towards significant of 0.05 < x ≤ 0.07 are *italicized*.

Compared to control conditions, chest compression increased mylohyoid EMG amplitude in males by 38% (t_22_ = -2.6, *p <* 0.05) and geniohyoid amplitude by 32% (t_22_ = -2.3, *p <* 0.05); there were no significant changes in females (Table 1).

### Vagotomy

Fig 1a and 1c show examples of the changes in swallow-related EMG activity following vagotomy (Table 3a). In males, mylohyoid EMG activity increased by 56% and geniohyoid increased by 57% (t_3_ = -11.1, *p* = 0.002, t_3_ = -9.4, *p* = 0.003, respectively); in females, geniohyoid amplitude increased by 51% (t_4_ = -2.4, *p* = 0.07), but this increase was not significant. Bilateral extra-thoracic vagotomy produced no change in swallow number, duration, or swallow-breathing coordination (Table 4a).

**Table 3:** Means, Standard Deviation (SD), *p*-values, and direction of change for swallow parameters during conditions of control (no feedback modulation or chest compression), feedback modulation alone (e.g. vagotomy), chest compression alone (without feedback modulation), and Chest Compression (CC) during feedback modulation conditions for both male and female groups. The left half of the table shows data comparing control conditions (no feedback modulation) to conditions adding vagotomy (A), nebulized lidocaine (B), or pleural injection of lidocaine (C), while the right half compares chest compression with the addition of each intervention. Amplitude is normalized to maximum of control and shown as a percentage.

**A**		**Control mean (SD)**	**Vagotomy mean (SD)**	***p*-value**	**Change**	**C****hest** **C****ompression mean (SD)**	**CC+Vagotomy mean (SD)**	***p*-value**	**Change**
	**Male (*n* = 6)**								
	Swallow Duration (ms)	286 (49)	307 (84)	0.41	**-**	303 (69)	325 (82)	0.34	**-**
	Swallow Number	4 (2)	4 (3)	0.93	**-**	4 (3)	3 (2)	**0.01**	**↓**
	Mylohyoid Amplitude (% max)	79 (13)	135 (20)	**0.002**	**↑**	110 (11)	153 (35)	0.12	**-**
	Geniohyoid Amplitude (% max)	86 (7)	143 (15)	**0.003**	**↑**	110 (16)	160 (22)	**0.05**	**↑**
	**Female (*n* = 6)**								
	Swallow Duration (ms)	300 (69)	311 (69)	0.72	**-**	280 (25)	322 (64)	0.29	**-**
	Swallow Number	7 (6)	5 (6)	0.34	**-**	6 (6)	3 (4)	*0*.*06*	↓
	Mylohyoid Amplitude (% max)	74 (18)	130 (101)	0.22	**-**	84 (15)	243 (188)	0.19	**-**
	Geniohyoid Amplitude (% max)	70 (23)	121 (69)	*0*.*07*	↑	86 (15)	163 (80)	0.13	**-**
**B**		**Control mean (SD)**	**Nebulize mean (SD)**	***p*-value**	**Change**	**C****hest** **C****ompression mean (SD)**	**CC+Nebulize mean (SD)**	***p*-value**	**Change**
	**Male (*n* = 6)**								
	Swallow Duration (ms)	246 (52)	204 (41)	0.12	**-**	244 (37)	198 (41)	**0.02**	**↓**
	Swallow Number	6 (2)	3 (3)	0.08	**-**	5 (3)	2 (2)	**0.03**	**↓**
	Mylohyoid Amplitude (% max)	61 (25)	88 (82)	0.44	**-**	122 (54)	105 (78)	0.69	**-**
	Geniohyoid Amplitude (% max)	82 (10)	108 (24)	0.14	**-**	102 (37)	119 (31)	0.19	**-**
	Thyroarytenoid Amplitude (% max)	86 (15)	70 (26)	0.28	**-**	100 (49)	140 (161)	0.53	**-**
	**Female (*n* = 3)**								
	Swallow Duration (ms)	314 (57)	194 (58)	0.20	**-**	342 (60)	217 (45)	**0.01**	**↓**
	Swallow Number	9 (10)	5 (6)	0.23	**-**	10 (6)	1 (0)	0.13	**-**
	Mylohyoid Amplitude (% max)	76 (22)	48 (8)	0.10	**-**	82 (26)	54 (30)	0.12	**-**
	Geniohyoid Amplitude (% max)	76 (23)	40 (18)	*0*.*07*	↓	81 (22)	41 (28)	*0*.*70*	↓
	Thyroarytenoid Amplitude (% max)	89 (13)	60 (46)	0.40	**-**	103 (19)	77 (63)	0.50	**-**
**C**		**Control mean (SD)**	**Pleural Injection mean (SD)**	***p*-value**	**Change**	**C****hest** **C****ompression mean (SD)**	**CC+Pleural Injection mean (SD)**	***p*-value**	**Change**
	**Male (*n* = 8)**								
	Swallow Duration (ms)	356 (69)	300 (112)	*0*.*06*	↓	359 (68)	307 (78)	0.11	**-**
	Swallow Number	9 (6)	6 (3)	0.15	**-**	6 (5)	5 (3)	0.42	**-**
	Mylohyoid Amplitude (% max)	71 (16)	41 (23)	**0.01**	**↓**	76 (39)	64 (37)	0.51	**-**
	Geniohyoid Amplitude (% max)	66 (19)	63 (48)	0.81	**-**	81 (23)	78 (52)	0.89	**-**
	Thyroarytenoid Amplitude (% max)	85 (9)	78 (31)	0.56	**-**	100 (13)	86 (38)	0.26	**-**
	**Female (*n* = 6)**								
	Swallow Duration (ms)	278 (55)	215 (37)	0.13	**-**	281 (42)	230 (47)	0.08	**-**
	Swallow Number	11 (7)	7 (8)	0.11	**-**	7 (8)	7 (7)	0.51	**-**
	Mylohyoid Amplitude (% max)	60 (6)	41 (12)	**0.03**	**↓**	60 (26)	45 (25)	0.34	**-**
	Geniohyoid Amplitude (% max)	69 (9)	44 (10)	**0.02**	**↓**	71 (26)	51 (27)	0.35	**-**
	Thyroarytenoid Amplitude (% max)	82 (9)	67 (25)	0.23	**-**	98 (23)	75 (18)	*0*.*07*	↓

Reported *p*-values are from Student’s paired t-test. Significance is bolded at *p*-values ≤ 0.05 and trending *p*-values of 0.05 < x ≤ 0.07 are *italicized*.

**Table 4:** Number of swallows during each phase of breathing during conditions of control (no feedback modulation or chest compression), feedback modulation alone (e.g. vagotomy), chest compression alone (without feedback modulation), and Chest Compression (CC) during feedback modulation conditions for both male and female groups. The left half of the table shows data comparing control conditions (no feedback modulation) to conditions adding vagotomy (A), nebulized lidocaine (B), or pleural injection of lidocaine (C), while the right half compares chest compression with the addition of each intervention.

**A**		**Control # of Swallows**	**Vagotomy # of Swallows**	***p*-value**	**Z**	**C****hest** **C****ompression # of Swallows**	**CC+Vagotomy # of Swallows**	***p*-value**	**Z**
	**Male**			0.13	-1.51			0.16	-1.41
	Inspiration (I)	1	0			0	0		
	Yield	8	5			9	2		
	Late Expiration (Late E)	16	19			14	13		
	**Female**			0.71	-0.38			**0.01**	-2.53
	Inspiration (I)	0	0			0	0		
	Yield	18	11			15	1		
	Late Expiration (Late E)	26	17			23	18		
**B**		**Control # of Swallows**	**Nebulize # of Swallows**	***p*-value**	**Z**	**C****hest** **C****ompression # of Swallows**	**CC+Nebulize # of Swallows**	***p*-value**	**Z**
	**Male**			*0*.*06*	-1.9			0.56	-0.58
	Inspiration (I)	1	0			3	3		
	Yield	23	10			8	7		
	Late Expiration (Late E)	24	9			23	10		
	**Female**			0.16	-1.41			0.32	-1.00
	Inspiration (I)	2	0			1	0		
	Yield	7	1			2	1		
	Late Expiration (Late E)	27	15			31	2		
**C**		**Control # of Swallows**	**Pleural Injection # of Swallows**	***p*-value**	**Z**	**C****hest** **C****ompression # of Swallows**	**CC+Pleural Injection # of Swallows**	***p*-value**	**Z**
	**Male**			0.23	-1.21			0.30	-1.03
	Inspiration (I)	2	2			5	5		
	Yield	18	10			8	11		
	Late Expiration (Late E)	61	37			48	20		
	**Female**			0.48	-0.71			**0.008**	-2.65
	Inspiration (I)	1	0			1	0		
	Yield	37	20			11	9		
	Late Expiration (Late E)	51	24			51	20		

Reported *p*-values are from Wilcoxon signed ranks test. Significance is **bolded** at *p*-values < 0.05 and *p*-values of 0.05 < x < 0.07 are *italicized*.

When compared to chest compression alone, the addition of bilateral vagotomy (Table 3a) significantly decreased swallow number in males (4 ± 3 to 3 ± 2; t_5_ = 4.0 *p <* 0.010), and increased geniohyoid EMG activity (t_4_ = -3.2, *p <* 0.049). In females, vagotomy produced a trend toward reduction in swallow number (6 ± 6 to 3 ± 4; t5 = 2.4 p < 0.063). Vagotomy caused a significant change in swallow-breathing coordination in female animals only (Fig 2c), with 95% of swallows (18 of 19) occurring during late E (z = -2.5, *p* = 0.011; Table 4a).

### Local anesthesia of PSRs via nebulized lidocaine inhalation

When compared to chest compression alone, the addition of lidocaine nebulization (Table 3b) significantly decreased swallow number in males (from 5 ± 3 to 2 ± 2; t_5_ = 2.9, *p* = 0.033), but produced no change in females. This intervention produced no significant changes in swallow-breathing coordination (Table 4b) in either sex. However, swallow duration was reduced in both male (244 ± 37ms to 198 ± 41ms; t_4_ = 4.0, *p* = 0.014) and female (342 ± 60 to 217 ± 45ms; t_2_ = 8.5, *p* = 0.014) groups, but there were no accompanying significant effects in EMG amplitudes of swallow-related muscles.

### Local anesthesia of pleural afferents via lidocaine injection

We locally anesthetized pleural afferents by injecting lidocaine into the pleural space (Table 3c). These injections caused significant decreases in swallow-related mylohyoid amplitude in both males (-30%; t_7_ = 3.6, *p* = 0.01) and females (-19%; t_4_ = 3.4, *p* = 0.027). Additional significant decreases in the geniohyoid amplitudes were also seen in females (-25%; t_4_ = 3.6, *p* = 0.023) (Fig 1b and 1d).

When compared to chest compression alone (Table 3c), the addition of lidocaine injections produced a significant change in swallow-breathing coordination in female animals (Fig 2d), with 69% of swallows (20 of 29) occurring in late E and 31% (9 of 29) during yield. These changes represent a significant shift to late E (Z = -2.65, *p* = 0.008; Table 4c). In females, thyroarytenoid amplitude was reduced by 23%, but this was non-significant (t_4_ = 2.5, *p* = 0.07).

## Discussion

This is the first study to investigate the effects of both vagal and spinal afferent feedback on swallow-breathing coordination in the rat. Our results suggest that there are major sex differences in swallow-breathing coordination, and that disrupting vagal feedback produces effects distinct from those seen with disruption of pleural spinal feedback. Male animals appear to rely more on PSR-mediated volume feedback, while alterations in spinal feedback produced greater effects in female animals. Our results confirm that both vagal and non-vagal afferent feedback sources are necessary for ensuring a stable swallow motor pattern in the rat.

### Sex differences in swallow-breathing coordination

Following disruption of PSRs in male animals, swallow occurrence decreased. For female animals, swallow occurrence shifted to late expiration when chest compression alone was applied, and also when vagal feedback or pleural spinal feedback was reduced (by vagotomy or pleural lidocaine injections, respectively) during chest compression.

In humans, both male and female, swallow timing is dependent on lung volume [27, 29, 31], which has been attributed to volume-related feedback via activation of PSRs. This is consistent with our results in the male rodents. Nebulization of lidocaine disrupted volume feedback. When combined with chest compression, which forces operation at lower lung volumes, swallow occurrence decreased. This suggests that PSR feedback is involved in swallow occurrence, consistent with our previous observation that swallow is inhibited at low lung volumes [27]. Swallows that occur during the inspiratory phase are presumed to increase aspiration risk [24, 61], which we hypothesize may be due to a mechanical advantage of bolus movement from an area of high pressure (pharynx) to an area of low pressure (esophagus). The current results are consistent with our previous theory that lung volume is a major factor in swallow-breathing phase preference [27]. The current results are also consistent with previous studies which demonstrated that a majority of swallows occur during expiration [14]. When volume feedback is reduced, or when transdiaphragmatic pressure is disrupted by laparotomy [46], swallow phase preference begins to move away from the classically predominant expiration phase and shifts toward the inspiration-to-expiration transition phase.

Considering that swallow-breathing coordination in females was altered only under chest compression conditions in the current study, we hypothesize that chest wall proprioception is the dominant feedback source in female rats. In addition to direct monitoring by PSRs, thoracic stretch receptors indirectly monitor lung volume [62] by detecting changes in muscle length and tension [63, 64]. During chest compression conditions, swallows retained an expiratory preference, even when we altered vagal and pleural spinal afferent feedback. In our complementary study [55], chest compression prolonged late expiration duration in female rats. The dominance of swallow during late expiration could be attributed to the large proportion of the respiratory cycle that is spent in expiration, which would ensure adequate time for swallow to occur in safe conditions [14].

Female rats appear to rely more on the contribution of thoracic movements for breathing, in contrast to male animals, who appear to rely more on movement of the diaphragm [65–67]. Compared to males, females also have a smaller ratio of lung volume to body mass [68] and a smaller rib cage [65]. Considering that we used the same chest band for all experiments—it was not sized relative to the different chest wall sizes of male and female animals—chest compression could have been greater for the females than males. Other physiological sex differences, such as hormones, also might influence swallow-breathing coordination.

### Vagal and spinal feedback influences on upper airway muscle amplitude during swallow

When PSR activity is experimentally reduced, upper airway tone is increased in the cat and dog [69, 70]. When we reduced PSR feedback by nebulizing lidocaine, we saw no change in upper airway muscle activity, however, when we removed PSR activity by bilateral vagotomy, swallow-related upper airway muscle activity increased, likely due to disinhibition [71]. Nebulized lidocaine may have affected other afferents in the airway mucosa (in addition to PSRs) by selectively inhibiting or stimulating particular airway vagal afferent types, while vagotomy would eliminate all vagal sensory information. Lidocaine injected into the pulmonary circulation selectively inhibits airway mechanosensors (rapidly adapting and slowly adapting (PSR) receptors) but concurrently stimulates airway chemosensors (C-fiber and high threshold Aδ-receptors) [72]. Such possible differential effects of lidocaine on distinct vagal afferents may explain the differences we saw in the results of nebulized lidocaine compared to those of bilateral vagotomy. When we perturbed spinal feedback by injecting lidocaine into the pleural space, upper airway muscle activity decreased, suggesting that pleural spinal afferents provide excitatory modulation of upper airway muscle activity during swallow. Together, these results indicate that mechanisms mediated by both vagal and spinal afferent feedback are important for the regulation of larger motor units during swallow (defined by alterations in EMG amplitude). Furthermore, since swallow amplitude was modulated when vagal or pleural spinal feedback was perturbed, we propose that vagal/spinal afferent input balance is required for normal swallow behavior.

### Swallow duration relies on both vagal and spinal feedback

In the condition of chest compression, when nebulized lidocaine was added to further reduce PSR feedback, swallow duration was decreased in both male and female animals. As volume feedback from both vagal and proprioceptive spinal sources appear to be important for swallow, pharmacologically and mechanically reducing PSR feedback by nebulizing lidocaine during chest compression would increase the risk of dysfunctional swallow. In this case, swallows may occur more quickly to maintain airway patency [14]. The decrease in swallow duration that we observed could result from an underlying decrease in central swallow excitability, but this is unlikely, considering that swallow number and amplitude were unchanged.

### EMG amplitude and duration are not correlated

The results of this study further support our hypothesis that there are different central mechanisms for regulating swallow amplitude and duration. Clinically, it has been assumed that swallow duration positively correlates with force production, as defined by swallow phase relationships in videofluoroscopy exams [13]. We have now established that swallow-related EMG amplitude and duration are not correlated in cats [13, 46, 73], humans [74, 75], or rats (present study). The inability to assess this using visual metrics (videofluoroscopy and endoscopy) supports the need for development of “strength” related clinical metrics in order to better investigate this property of swallow pattern generation.

### Limitations

The data cohort of females in which lidocaine was nebulized is small, due in part to a high number of animal deaths from cardio-respiratory failure. This cohort originally consisted of 8 females, all of varying weights and estrus cycles, of which only 3 survived the protocol. Of note, based on anesthetic dose and response to noxious stimulation, male and female rats were at equivalent anesthetic states. We speculate that the females were more sensitive to the nebulized lidocaine. General anesthesia also introduces potential limitations due to effects of sodium pentobarbital on gamma motoneurons [76]. The dampening effects of this anesthetic may have reduced proprioceptive feedback in our study.

## Conclusion

Our results provide evidence that, while the swallow central pattern generator is located in the brainstem, perturbations of peripheral feedback can also disrupt swallow in predictable ways. This study adds to the body of evidence demonstrating that swallow-breathing coordination is dependent upon lung volume. This has potential clinical implications, as development of therapies targeting specific lung volumes to allow for safe swallowing would benefit patient populations for whom swallow is a risky behavior, including patients with cervical and thoracic spinal cord injuries.

## Supporting information

S1 Data (XLSX)
